# rs6971 TSPO polymorphism in Parkinson's disease

**DOI:** 10.1002/mds.70105

**Published:** 2025-11-03

**Authors:** Bina Patel, Marta Camacho, Jonathan R. Evans, David P. Breen, Thomas Foltynie, Sarah L. Mason, Gemma Cummins, Ruwani Wijeyekoon, Roger A. Barker, Caroline Helen Williams‐Gray

**Affiliations:** ^1^ John van Geest Centre for Brain Repair, Department of Clinical Neurosciences University of Cambridge Cambridge UK; ^2^ Cambridge University Hospitals NHS Foundation Trust Cambridge UK; ^3^ Nottingham University Hospital NHS Trust Nottingham UK; ^4^ Anne Rowling Regenerative Neurology Clinic, Institute for Neuroscience and Cardiovascular Research University of Edinburgh Edinburgh UK; ^5^ Usher Institute of Population Health Sciences and Informatics University of Edinburgh Edinburgh UK; ^6^ Department of Movement and Clinical Neurosciences UCL Institute of Neurology, Queen Square London UK; ^7^ Cambridge and Peterborough NHS Foundation Trust Cambridge UK

Mitochondrial translocator protein (TPSO) positron emission tomography (PET) imaging is widely used to study neuroinflammation in vivo. TSPO, located on the outer mitochondrial membrane, is upregulated in microglia in association with neuroinflammation,^1^ with increased binding observed in Parkinson's disease (PD).^2^ The first‐generation TSPO ligand [^11^C]PK11195 has important limitations due to low signal‐to‐noise ratio, and a high production failure rate. Second‐generation TSPO ligands (eg, [^11^C]PBR28/[^18^F]DPA714) offer improved binding affinity, specificity, and bioavailability,^3^ making them valuable in clinical studies as an index of brain inflammation. Since chronic neuroinflammation emerges early in PD and potentially drives disease progression,^4^ TSPO‐PET imaging can help evaluate the effectiveness of anti‐inflammatory therapies and support the development of disease‐modifying therapies.

However, second‐generation TSPO ligands are sensitive to a single nucleotide polymorphism in the TSPO gene (rs6971 Ala147Thr) producing high‐ (HAB), mixed‐ (MAB) and low‐ (LAB) affinity binders.^5^ LABs, (approximately 10% of Europeans^6^), have lower signal‐to‐noise ratios, challenging scan interpretation, often leading to their exclusion from studies utilizing these ligands, potentially introducing selection bias and limiting generalizability.

A previous PD study reported that the rs6971 polymorphism did not affect baseline demographics but HABs progressed to dyskinesia faster.^7^ This study was limited by a relatively small sample size (n = 150) and short follow‐up period. We aimed to further explore the impact of TSPO genotype on disease progression and clinical outcomes in PD.

393 participants (98.5% Caucasian) with idiopathic PD from two incident population‐representative cohorts: Parkinsonism: Incidence and Cognitive Heterogeneity in Cambridgeshire (PICNICS) and Cambridgeshire Parkinson's Incidence from GP to Neurologist (CamPaIGN) were genotyped and underwent at least 12 years of follow‐up with regular clinical assessments (Supplementary Methods). The frequencies of HABs (46.6%), MABs (44.8%), and LABs (8.6%) matched prior European data.^6^ No statistical difference was seen between the genotypic groups in terms of baseline characteristics including sex, age at diagnosis, levodopa equivalent daily dose (LEDD), Beck Depression Inventory (BDI), Mini Mental State Examination (MMSE), and Movement Disorder Society Unified Parkinson's Disease Rating Scale‐Part III (MDS‐UPDRS‐III) scores (Table S1).

Kaplan–Meier survival analysis showed no difference in progression to dyskinesia, dementia, postural instability, or death between the genotypic groups (Fig. 1). Cox proportional hazard regression models confirmed that rs6971 was not a significant predictor of these outcomes (Table S2).

**FIG. 1:**
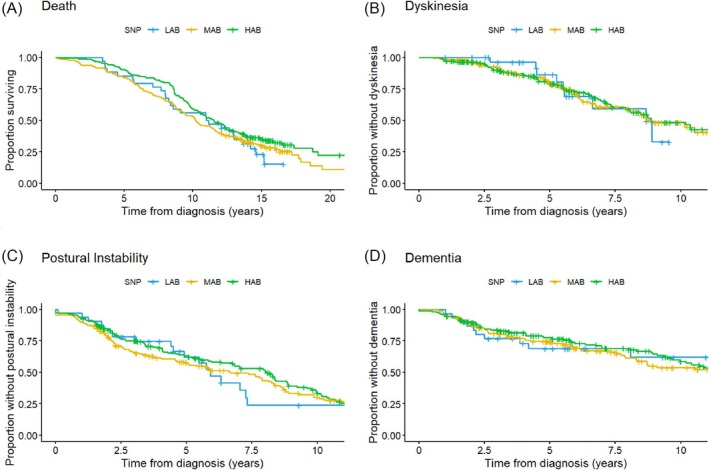
Kaplan–Maier survival curves for time to reach negative outcomes: death (A), dyskinesia (B), postural instability (C), and dementia (D). SNP, single nucleotide polymorphism; LAB, low‐affinity binders; MAB, mixed‐affinity binders; HAB, high‐affinity binders. [Color figure can be viewed at wileyonlinelibrary.com]

Line plots with locally estimated scatterplot smoothing (LOESS) were used to visualize longitudinal changes in MDS‐UPDRS‐III, MMSE, and Addenbrookes Cognitive Examination Revised (ACE‐R) scores between the genotypic groups (Fig. S1). Linear mixed‐effects models of longitudinal MDS‐UPDRS‐III and MMSE scores confirmed that rs6971 was not a predictor of motor or cognitive progression and was the first variable removed via backward selection (Tables [Link], [Link]). The MMSE model demonstrated a poor fit due to ceiling effects and non‐normal data distribution. An alternative model using ACE‐R scores also found no significant effect of rs6971 on cognitive decline (Table S5).

Our results indicate that the rs6971 genetic polymorphism is not associated with baseline clinical characteristics or disease progression in PD, supporting the continued exclusion of LABs from studies using second‐generation TSPO‐PET ligands without compromising generalizability.

## Author Roles

(1) Research Project: A. Conception, B. Organization, C. Execution, D. Genotyping, E. Clinical Data Collection, F. Supervision; (2) Statistical Analysis: A. Design, B. Execution, C. Review and Critique; (3) Manuscript Preparation: A. Writing of the First Draft, B. Review and Critique.B.P.: 1A, 1D, 1E, 2B, 3A, 3B.

M.C.: 1E, 3B.

J.R.E.: 1E, 3B.

D.P.B.: 1E, 3B.

T.F.: 1E, 3B.

S.L.M.: 1E, 3B.

G.C.: 1E, 3B.

R.W.: 1E, 3B.

R.A.B.: 1E, 3B.

C.H.W.‐G.: 1E, 1F, 3B.

**Financial Disclosures for the Previous 12 Months**

The authors declare that there are no additional disclosures to report.

## Supporting information


**Data S1.** Supplementary Methods.

**Figure S1:** Line plots displaying Movement Disorder Society Unified Parkinson's Disease Rating Scale‐Part III (MDS‐UPDRS‐III) scores (A) Mini‐Mental State Examination (MMSE) scores (B), and Addenbrookes Cognitive Examination Revised (ACE‐R) (C) in participants by genotype group with locally estimated scatterplot smoothing (LOESS) to highlight longitudinal trends.

**Table S1:** Baseline demographic and clinical characteristics of Parkinson's disease patients stratified by mitochondrial translocator protein (TSPO) genotype.

**Table S2:** Results from four Cox proportional hazards regression models examining hazard ratios (HRs) for time to: (1) death, (2) dementia, (3) postural instability, and (4) dyskinesia.

**Table S3:** Summary of linear mixed‐effects model (LMEM) assessing predictors of change in Movement Disorder Society Unified Parkinson's Disease Rating Scale‐Part III (MDS‐UPDRS‐III) (motor scores) over time.

**Table S4:** Summary of linear mixed‐effects model (LMEM) assessing predictors of change in Mini‐Mental State Examination (MMSE) scores over time.

**Table S5:** Summary of linear mixed‐effects model (LMEM) assessing predictors of change in Addenbrookes Cognitive Examination Revised (ACE‐R) scores over time, PICNICs cohort only.

## Data Availability

The data that support these findings are available on request from the authors subject to negotiation of data sharing agreements.
