# Supplementary material for: rs6971 TSPO polymorphism in Parkinson's disease
Source: Mov Disord. 2025 Nov 3;41(2):541–3. doi: 10.1002/mds.70105 (PMC12951254; doi:10.1002/mds.70105)
Supplement: Supplementary file 1 — Data S1. Supplementary Methods. [file MDS-41-541-s001.docx]

**Supplementary Methods**

**Methods**

All patients were diagnosed with idiopathic PD using UK Parkinson’s Disease Society Brain Bank criteria. We included clinical data from two incident cohorts, recruited within two years of diagnosis: Parkinsonism: Incidence and Cognitive Heterogeneity in Cambridgeshire (PICNICS, n=280) and *Cam*bridgeshire *Pa*rkinson's *I*ncidence from *G*P to *N*eurologist (CamPaIGN, n=140) ^1^. To date, PICNICS participants have been followed up every 18 months for up to 12 years and CamPaIGN participants have been followed up every 2 years for up to 20 years. All data from the PICNICS cohort were used in this analysis, however, for the CamPAIGN cohort, only data up to 12 years of follow up were used to ensure consistency when combining the datasets. Mortality data were included for both cohorts up to a cutoff date of January 2025.

Participants underwent motor, cognitive and neuropsychological assessments at each research visit, including the Unified Parkinson’s Disease Rating Scale (UPDRS) in CamPaIGN or Movement Disorders Society Unified Parkinsons disease Rating Scale (MDS-UPDRS) in PICNICS, the Mini Mental State Exam (MMSE), the Addenbrookes Cognitive Examination Revised (PICNICs only), Levodopa equivalent daily dose (LEDD), Beck Depression Inventory (BDI) and Parkinson’s Disease Questionnaire 39 (PDQ-39). The National Adult Reading Test (NART) was used to estimate verbal IQ at baseline. UPDRS scores were converted to MDS-UPDRS scores using previously validated methods^2^. The number of comorbidities at baseline was quantified in terms of the number of organ systems affected using the Cumulative Illness Rating Scale (CIRS)^3^. Further information on the assessments used in each cohort can be found in previous publications^1,4,5^.

Blood was collected and genomic DNA was extracted at baseline using standard phenol/chloroform techniques. Genotyping was carried out with the TAQMAN thermofisher genotyping kit (C___2512465_20) for rs6971, following the manufacturer’s instructions using the Thermofisher QuantStudio Real-Time PCR Systems machine. Allelic discrimination plots were created in Quantstudio software.

All statistical analysis was completed in R studio. Kruskal Wallis tests were used for comparison of continuous baseline variables and χ2 tests for categoric variables between the three genotypic groups.

Progression to mortality and key disease milestones (dementia, dyskinesia, postural instability) was assessed using Kaplan–Meier survival analysis, where the time of PD diagnosis was t=0. For time to dementia, dyskinesia and postural instability withdrawals were censored at the time of last clinical assessment. Time to dyskinesia, dementia and postural instability was estimated as the midpoint between the visit when the outcome was first noted and the previous assessment.

Cox proportional hazard regression models were fitted to the entire cohort, using a backward stepwise approach based on Akaike Information Criterion (AIC), to evaluate whether there were associations between genotype and each outcome. Similarly, linear mixed effects models were used to evaluate associations between genotype and motor (MDS-UPDRS motor score) and cognitive (MMSE/ACE-R) progression over 12 years of follow up. All models were adjusted for clinically relevant baseline covariates (age, sex, verbal IQ, CIRS, MDS-UPDRS motor scores, BDI, LEDD and MMSE).

References:

1. Evans JR, Cummins G, Breen DP, et al. Comparative epidemiology of incident Parkinson’s disease in Cambridgeshire, UK. *Journal of Neurology, Neurosurgery and Psychiatry*; 87. Epub ahead of print 2016. DOI: 10.1136/jnnp-2015-312581.

2. Goetz CG, Stebbins GT, Tilley BC. Calibration of unified Parkinson’s disease rating scale scores to Movement Disorder Society‐unified Parkinson’s disease rating scale scores. *Movement Disorders* 2012; 27: 1239–1242.

3. LINN BS, LINN MW, GUREL L. CUMULATIVE ILLNESS RATING SCALE. *J Am Geriatr Soc*; 16. Epub ahead of print 1968. DOI: 10.1111/j.1532-5415.1968.tb02103.x.

4. Williams-Gray CH, Mason SL, Evans JR, et al. The CamPaIGN study of Parkinson’s disease: 10-year outlook in an incident population-based cohort. *J Neurol Neurosurg Psychiatry* 2013; 84: 1258–1264.

5. Stoker TB, Camacho M, Winder-Rhodes S, et al. Impact of GBA1 variants on long-term clinical progression and mortality in incident Parkinson’s disease. *J Neurol Neurosurg Psychiatry* 2020; 91: 695–702.
