# Supplementary material for: rs6971 TSPO polymorphism in Parkinson's disease
Source: Mov Disord. 2025 Nov 3;41(2):541–3. doi: 10.1002/mds.70105 (PMC12951254; doi:10.1002/mds.70105)
Supplement: Supplementary file 2 — Figure S1: Line plots displaying Movement Disorder Society Unified Parkinson's Disease Rating Scale‐Part III (MDS‐UPDRS‐III) scores (A) Mini‐Mental State Examination (MMSE) scores (B), and Addenbrookes Cognitive Examination Revised (ACE‐R) (C) in participants by genotype group with locally estimated scatterplot smoothing (LOESS) to highlight longitudinal trends. [file MDS-41-541-s002.docx]

| **A**  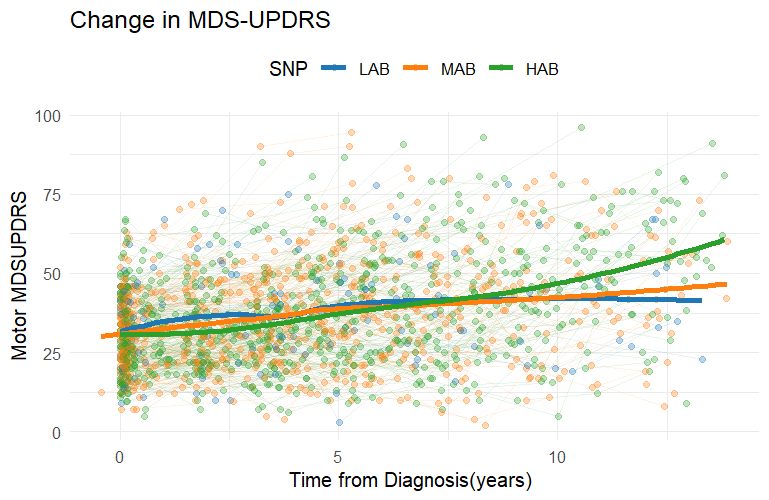 |
| --- |
| **B**  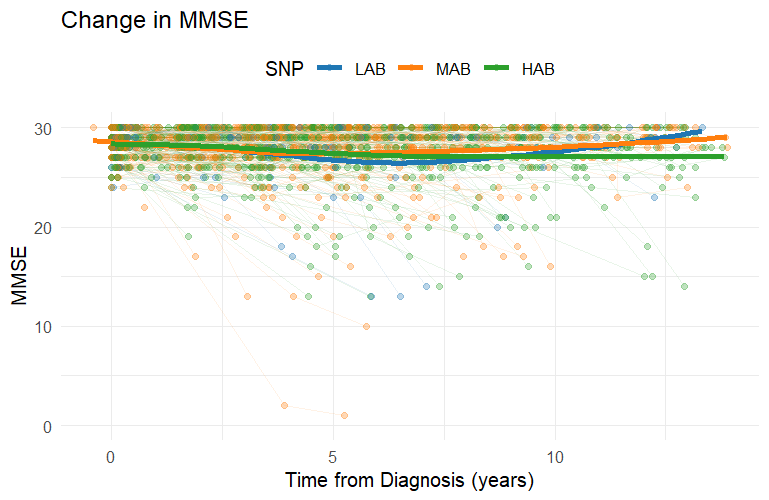 |
| C  **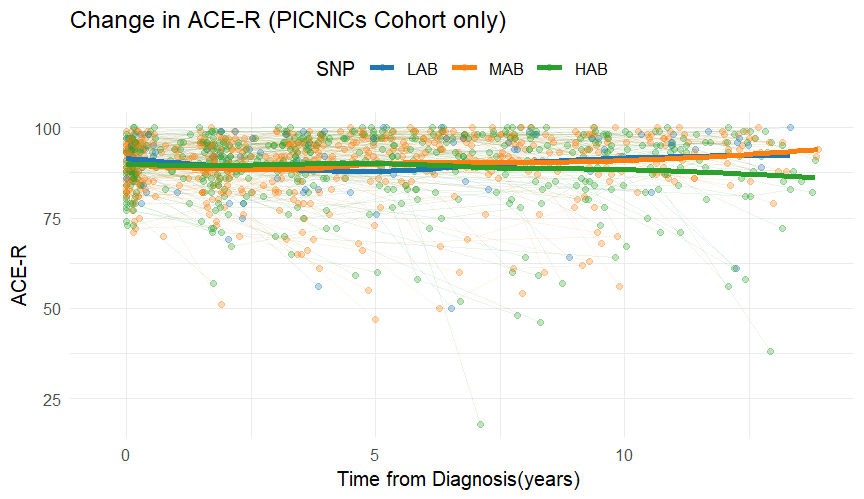** |
| **Supplementary Figure 1**: Line plots displaying MDS-UPDRS part III scores (A) MMSE scores (B) and ACE-R (C) in participants by genotype group with LOESS smoothing to hightlight longitudinal trends.  **Single Nucleotide Polymorphism (SNP). Low affinity binders (LAB), mixed affinity binders (MAB) high affinity binders (HAB); LOESS – locally estimated scatterplot smoothing** |
