# Supplementary material for: rs6971 TSPO polymorphism in Parkinson's disease
Source: Mov Disord. 2025 Nov 3;41(2):541–3. doi: 10.1002/mds.70105 (PMC12951254; doi:10.1002/mds.70105)
Supplement: Supplementary file 3 — Table S1: Baseline demographic and clinical characteristics of Parkinson's disease patients stratified by mitochondrial translocator protein (TSPO) genotype. [file MDS-41-541-s007.docx]

| **Supplementary Table 1**: **Baseline demographic and clinical characteristics of PD patients stratified by TSPO genotype**. | | | | |
| --- | --- | --- | --- | --- |
|  | **LAB**  **(n=34, 8.7%)** | **MAB**  **(n=176, 44.8%)** | **HAB**  **(n=183, 46.66%)** | **P Value** |
| Age (years) | 69.5 ± 8.42 (42-81.6) | 69.8 ± 10 (37-89.6) | 68.1 ± 9.25 (40.4-96.4) | 0.086 |
| Sex (% male) | 70.6 | 63.6 | 55.2 | 0.116 |
| Time from diagnosis (years) | 0.213 ± 0.218 (0-0.917) | 0.247 ± 0.344 (0-2.35) | 0.278 ±0.371 (0-1.88) | 0.908 |
| MMSE | 28.4 ± 1.37 (24-30) | 28.5 ± 1.52 (22-30) | 28.4 ± 1.44 (24-30) | 0.652 |
| MDS-UPDRS Part III | 31.6 ± 12.8 (9-59.3) | 31.9 ± 12.9 (7-71.9) | 30.8 ± 12.7 (5-67) | 0.663 |
| BDI | 6.97 ± 4.79 (0-20) | 7.65 ± 5.39 (0-26) | 7.25 ± 5.92 (0-28) | 0.474 |
| LEDD | 177 ± 203 (0-840) | 145 ± 181 (0-960) | 146 ± 192 (0-1040) | 0.453 |
| CIRS score | 2.12 ± 1.77 (0-7) | 2.08 ±1.49 (0-6) | 2.03 ±1.41 (0-6) | 0.919 |
| ACE-R* | 91.4 ± 5.35 (79-98) | 89.7 ± 6.36 (70-99) | 90.4 ± 6.01 (73-100) | 0.652 |
| Values shown as mean ± standard deviation (SD) (minimum and maximum values). Group means were compared using Kruskal Wallis tests. LAB- Low affinity binders; MAB - mixed affinity binders; HAB - high affinity binders; MMSE- Mini Mental State Examination; MDS-UPDRS – Movement Disorder Society Unified Parkinson’s Disease Rating Scale; BDI – Beck Depression Scale; LEDD – Levodopa equivalent daily dose, ACE-R – Addenbrookes Cognitive Examination Revised, *PICNICs cohort only, (LAB n=20, MAB n=121, HAB n=115) | | | | |
