# Supplementary material for: rs6971 TSPO polymorphism in Parkinson's disease
Source: Mov Disord. 2025 Nov 3;41(2):541–3. doi: 10.1002/mds.70105 (PMC12951254; doi:10.1002/mds.70105)
Supplement: Supplementary file 4 — Table S2: Results from four Cox proportional hazards regression models examining hazard ratios (HRs) for time to: (1) death, (2) dementia, (3) postural instability, and (4) dyskinesia. [file MDS-41-541-s005.docx]

| **Supplementary Table 2**: Results from four Cox proportional hazards regression models examining hazard ratios (HRs) for time to: (1) death, (2) dementia, (3) postural instability, and (4) dyskinesia. | | | | | | | | |
| --- | --- | --- | --- | --- | --- | --- | --- | --- |
| Outcome  Predictor | Death | | Dementia | | Postural Instability | | Dyskinesia | |
|  | HR (95% CI) | p-value | HR (95% CI) | p-value | HR (95% CI) | p-value | HR (95% CI) | p-value |
| MAB vs LAB | 1.09 (0.70–1.70)\* | 0.704 | 1.21 (0.59 – 2.47) | 0.602 | 1.16 (0.67–2.03) | 0.595 | 0.92 (0.43 – 1.99) | 0.840 |
| HAB vs LAB | 0.88 (0.57–1.37)\* | 0.601 | 1.31 (0.64 – 2.69) | 0.454 | 1.10 (0.63–1.93) | 0.742 | 0.88 (0.41 – 1.88) | 0.742 |
| Male vs Female | 0.56 (0.41–0.75)\* | <0.001*** | 0.76 (0.51 – 1.11) | 0.155 | 0.82 (0.61–1.12) | 0.210 | 1.34 (0.91 – 1.97) | 0.133 |
| Age at diagnosis | 1.11 (1.09–1.13)\* | <0.001*** | 1.07 (1.05 – 1.10) | <0.001*** | 1.06 (1.04–1.09) | <0.001*** | 0.98 (0.96 – 1.00) | 0.115 |
| Comorbidity score | 0.94 (0.86–1.04)\* | 0.210 | 0.97 (0.85 – 1.11) | 0.689 | 1.00 (0.90–1.11) | 0.980 | 0.95 (0.82 – 1.09) | 0.444 |
| LEDD | 1.00 (0.99–1.00)\* | 0.910 | 1.00 (0.999 – 1.001) | 0.939 | 1.00 (1.00–1.00) | 0.176 | 1.00 (1.00 – 1.00) | 0.280 |
| MDS-UPDRS Part III | 1.02 (1.01–1.03)\* | <0.001*** | 1.03 (1.01 – 1.05) | <0.001*** | 1.01 (1.00–1.02) | 0.086 | 1.02 (1.00 – 1.04) | 0.031* |
| MMSE | 0.91 (0.82–1.01)\* | 0.098 | 0.86 (0.73 – 1.00) | 0.053 | 0.91 (0.80–1.03) | 0.147 | 1.19 (0.99 – 1.42) | 0.070 |
| Verbal IQ | 1.00 (0.98–1.01)\* | 0.845 | 0.98 (0.96 – 1.00) | 0.031* | 0.99 (0.98–1.01) | 0.335 | 1.03 (1.01 – 1.06) | 0.015* |
| BDI | 1.01 (0.99–1.03)\* | 0.579 | 1.02 (0.99 – 1.05) | 0.292 | 1.02 (0.99–1.04) | 0.167 | 1.02 (0.99 – 1.06) | 0.255 |
| Low affinity binders (LAB), mixed affinity binders (MAB) high affinity binders (HAB); MMSE- Mini Mental State Examination; MDS-UPDRS – Movement Disorder Society Unified Parkinson’s Disease Rating Scale; BDI – Beck Depression Scale; LEDD – Levodopa equivalent daily dose. *** p<0.001, ** p <0.01, * p <0.05 | | | | | | | | |
