# Supplementary material for: rs6971 TSPO polymorphism in Parkinson's disease
Source: Mov Disord. 2025 Nov 3;41(2):541–3. doi: 10.1002/mds.70105 (PMC12951254; doi:10.1002/mds.70105)
Supplement: Supplementary file 5 — Table S3: Summary of linear mixed‐effects model (LMEM) assessing predictors of change in Movement Disorder Society Unified Parkinson's Disease Rating Scale‐Part III (MDS‐UPDRS‐III) (motor scores) over time. [file MDS-41-541-s003.docx]

| **Supplementary Table 3**: Summary of linear mixed-effects model (LMEM) assessing predictors of change in MDS-UPDRS ( Movement Disorder Society Unified Parkinson’s Disease Rating Scale) part III (motor scores) over time. | | | | |
| --- | --- | --- | --- | --- |
| Predictor | Non-standardised Estimate (β) | Standardised Estimate (β) | p value | Significance |
| SNP | -0.82 | -0.82 | 0.37 |  |
| Time from diagnosis | 2.17 | 7.68 | <0.001 | *** |
| Age at visit | 0.47 | 4.37 | <0.001 | *** |
| Sex (male = 0) | -3.06 | -3.06 | 0.01 | * |
| LEDD at visit | -0.004 | -1.83 | <0.001 | *** |
| Levodopa equivalent daily dose (LEDD), Single Nucleotide Polymorphism (SNP). All continuous variables except for the outcome, were standardised in standardised models. *** p<0.001, ** p <0.01, * p <0.05. | | | | |
