# Supplementary material for: rs6971 TSPO polymorphism in Parkinson's disease
Source: Mov Disord. 2025 Nov 3;41(2):541–3. doi: 10.1002/mds.70105 (PMC12951254; doi:10.1002/mds.70105)
Supplement: Supplementary file 6 — Table S4: Summary of linear mixed‐effects model (LMEM) assessing predictors of change in Mini‐Mental State Examination (MMSE) scores over time. [file MDS-41-541-s006.docx]

| **Supplementary Table 4:** Summary of linear mixed-effects model (LMEM) assessing predictors of change in MMSE (Mini-mental state examination) scores over time. | | | | |
| --- | --- | --- | --- | --- |
| Predictor | Non-standardised Estimate (β) | Standardised Estimate (β) | p value | Significance |
| SNP | -0.02 | -0.02 | 0.873 |  |
| Time from diagnosis | -0.34 | -1.22 | <0.001 | *** |
| Age at visit | -0.05 | -0.44 | <0.001 | *** |
| Sex | -0.01 | -0.01 | 0.967 |  |
| BDI at baseline | -0.02 | -0.12 | 0.086 | . |
| Verbal IQ at baseline | 0.06 | 0.60 | <0.001 | *** |
| Becks Depression Inventory (BDI), Single Nucleotide Polymorphism (SNP). All continuous variables except for the outcome, were standardised in standardised models. *** p<0.001, ** p <0.01, * p <0.05. | | | | |
