# Supplementary material for: rs6971 TSPO polymorphism in Parkinson's disease
Source: Mov Disord. 2025 Nov 3;41(2):541–3. doi: 10.1002/mds.70105 (PMC12951254; doi:10.1002/mds.70105)
Supplement: Supplementary file 7 — Table S5: Summary of linear mixed‐effects model (LMEM) assessing predictors of change in Addenbrookes Cognitive Examination Revised (ACE‐R) scores over time, PICNICs cohort only. [file MDS-41-541-s004.docx]

| **Supplementary Table 5:** Summary of linear mixed-effects model (LMEM) assessing predictors of change in ACE-R (Addenbrookes Cognitive Examination Revised) scores over time, PICNICs cohort only. | | | | |
| --- | --- | --- | --- | --- |
| Predictor | Non-standardised Estimate (β) | Standardised Estimate (β) | p value | Significance |
| SNP | 0.38 | 0.38 | 0.483 |  |
| Time from diagnosis | -0.79 | -2.99 | <0.001 | *** |
| Age at visit | -0.21 | -1.76 | <0.001 | *** |
| Sex | 0.48 | 0.48 | 0.498 |  |
| BDI at baseline | -0.04 | -0.23 | 0.514 |  |
| Verbal IQ at baseline | 0.42 | 3.87 | <0.001 | *** |
| Becks Depression Inventory (BDI), Single Nucleotide Polymorphism (SNP). All continuous variables except for the outcome, were standardised in standardised models. *** p<0.001, ** p <0.01, * p <0.05. | | | | |
